# Supplementary material for: Are carbon emissions trading and green financial instruments synergistic? -Comprehensive quantitative research based on content analysis
Source: PLoS One. 2024 Mar 7;19(3):e0298601. doi: 10.1371/journal.pone.0298601 (PMC10919601; doi:10.1371/journal.pone.0298601)
Supplement: S1 Data — (ZIP) [file pone.0298601.s001.zip › Data/地级市Z_F工作报告.zip!/╜¡╦╒/╨∞╓▌╩╨/└┤╫╘╣1⁄2╓┌║┼í╛┬φ┐╦╩2╛▌═°í┐_2009.docx]

各位代表：

　　现在，我代表市人民政府向大会作工作报告，请予审议，并请市政协各位委员和其他列席人员提出意见。

　　　　　　　　　　　　　　　　　　　　一、2008年工作回顾

　　2008年，是国内外宏观经济环境复杂多变、形势最不明朗的一年，是经济发展经受前所未有的严峻挑战和重大考验的一年，也是省委、省政府对徐州支持力度最大的一年。一年来，全市上下在省委、省政府和市委的正确领导下，解放思想，开拓创新，攻坚克难，拼搏进取，在全省发展的大格局中，我市经济和社会发展创下近年来最好水平。

　　（一）主动应对复杂局面，经济发展继续保持良好态势。去年以来，面对国际金融危机不断加深的严峻形势和国内经济紧运行的压力，我们积极研究和落实有效措施，主动应对外部挑战，抢占了保增长、促调整的先机。在工作中，始终坚持招商选资和项目建设不犹豫、不松劲，在下半年宏观调控政策尚未明朗的情况下，就未雨绸缪、及早动手，牢牢把握了重大项目推进的主动权；紧紧抓住省委、省政府实施振兴徐州老工业基地的战略契机，大力激发和调动广大干部群众的积极性、创造性，使全市经济在复杂形势和重重困难下，实现了平稳较快发展。全年地区生产总值完成2000亿元（预计，下同）、增长14%；财政一般预算收入达125.85亿元、增长25.4%；全社会固定资产投资完成1250亿元、增长30%，主要经济指标增幅全面高于全省平均水平。

　　在去年的经济发展中，最突出的特点就是打造形成了新能源这一新兴产业，工业经济结构调新、调高、调优工作取得重要进展。以中能硅业等企业为代表的光伏光电产业发展迅猛，我市一跃成为全国最大的多晶硅生产基地；以罗特艾德、维斯塔斯等企业为代表的风电产业蓬勃兴起。以工程机械和专用车辆为代表的装备制造业、以食品和农副产品加工为代表的食品产业、以煤盐化工和精细化工为代表的化工产业进一步发展壮大。全市规模以上工业增加值完成810亿元、增长19%。完成工业投资660.9亿元，徐工专用车技改、中能多晶硅三期、睢宁星星冰箱冰柜、新沂恒盛化肥20万吨低压甲醇、沛县丰源铝箔等63个亿元以上项目竣工投产；徐工筑路旋挖钻孔机、徐矿集团综合利用电厂等58个亿元以上项目开工建设；丰县建滔煤化工、铜山爱斯科耐磨件二期、邳州恒鑫化工二期、贾汪海通特钢等项目扎实推进。全市节能降耗和控污减排成效明显，高新技术产业产值大幅增长，工业经济发展速度、质量和效益同步提升。

　　在坚定不移地推进新型工业化的同时，坚持做强做优农业发展优势。不断夯实粮食生产基础，强化高效农业规模壮大和层次提升，粮食连续五年大幅增产，高效农业总面积、新增面积、占耕地面积比重三项指标，以及以县为单位的8个单项高效农业产业规模位居全省第一。农村各项基础设施建设加快推进，150个市级新农村示范村基本建成，农村经济和新农村建设成效突出。

　　进一步加大以生产性服务业为主的现代服务业发展力度，全市服务业呈现加快发展态势。服务业增加值和社会消费品零售总额分别增长14.5%和25%；中心商圈加速集聚，印尼力宝、苏宁综合商务广场、彭城北路商业广场等重大项目相继启动，核心区集聚企业达1060家，对淮海经济区的辐射带动力显著增强；各类专业市场加快发展，一批重点物流项目进展顺利，旅游经济各项指标均居苏北首位。

　　（二）体制机制不断创新，改革开放深入推进。各项改革扎实开展。骨干企业上市和资本运作步伐加快，徐工集团成功收购春兰汽车制造公司，恩华药业顺利上市，蓝丰生化等7家企业已进入上市实质性运作阶段。在基本完成国企改制任务的基础上，拨付1.9亿元妥善解决历史遗留问题，研究出台了一系列保护职工合法权益的政策措施。事业单位改革和乡镇机构改革试点工作稳步实施，市级机关公务员津补贴规范工作基本完成。民营经济快速发展，为全市经济注入新的活力。

　　开放型经济健康发展。去年下半年以来，不少外商的投资更趋谨慎甚至采取观望态度，在这种形势下，我们坚持招商选资不动摇，更加注重强化亲商、安商、富商工作，以产业链延伸引进项目，以招商方式创新吸引投资，有力推动了利用外资的增长和结构优化。全市实际到帐注册外资5.8亿美元、增长31%。外贸出口保持较快增长，出口总额达21.5亿美元、增长30%。新签外经合同额、完成营业额分别增长22%和23%。高层次人才培育和引进力度加大，95人入选省高层次创新创业人才培育计划，千名招商人才选拔培养工作进展顺利。

　　开发区主阵地作用日益凸显。徐州经济开发区强力推动“二次创业”，狠抓载体功能完善、体制机制创新和服务环境优化，在招大引强、产业集聚上取得重大突破，成功引进了凯迪环保等一批科技含量高、产业带动力强、综合效益好的大项目，实际到帐注册外资和自营出口在全市占比分别达65.7%和50%。其他7家省级开发区也保持较快增长势头，南北共建园区和特色园区建设取得新的进展。

　　（三）城市综合功能和环境质量明显提升，城市规划建设和管理水平跃上新台阶。把构建现代化大交通格局作为强化中心城市地位的首要任务，基本完成京沪高铁徐州段征地拆迁任务，全面启动了各项建设工作；丰沛铁路和宿新高速公路奠基开工，徐济高速公路、京杭运河主通道建设、淮西客运枢纽站改扩建、火车站综合改造等工程加快实施，观音机场被国务院正式批准为一类口岸；国内跨铁路最多的大桥——和平大桥建成通车，市区49条道路综合整治全面完成，环城路贯通、机场路改造等工程基本完工，奔腾大道西延、天齐路南延、黄河北路西延等工程进展顺利。

　　突出抓好功能性项目建设，努力克服资金、土地、拆迁等诸多困难，保持了老城改造和新城开发的强劲势头。老城区以音乐厅、美术馆等为代表的一批重大功能性项目相继建设，填补了中心城市的功能空白；新城区科技馆、档案馆、规划馆等功能性项目主体基本完工，学校、医院等配套设施项目陆续开工建设；高铁国际商务区建设的前期工作扎实推进。

　　坚持一手抓生态建设、一手抓环境保护，城乡环境质量进一步改善。精心规划建设了云龙公园、云龙山东坡运动广场、大龙湖西部近30万平方米生态湿地等一批精品园林和绿地。尤其是云龙公园改造建设工程开创我市大型综合性公园全敞开管理先河；云龙山东坡运动广场为广大群众开辟了融入自然、运动休闲的新场所；新城区市民广场和大龙湖绿化获得国家优秀园林绿化工程金奖，小南湖景观工程获江苏省人居环境范例奖。九里湖西湖、和平路绿化、白云山公园一期等工程全面完成，截污导流工程开工建设，城市环境空气质量良好天数达到320天，全市森林覆盖率和市区建成区绿化覆盖率分别达27%和40.5%。

　　着力向机制创新要管理绩效，城市管理机制有了重要转变。在市区道路、街巷保洁上实行了全方位市场化运作，基本实现保洁招标全覆盖；市区公共绿地实现市场招标养护管理；在城市亮化上推行了照明标准化、规范化管理。“严管街”整治成效明显，城市管理的规范化和精细化水平进一步提高。一年来，城市规划、建设和管理的强力推进与不断创新，使我市展现出更绿、更亮、更美的新形象。

　　（四）民生民计持续改善，和谐发展成效显著。坚持实施积极主动的创业和就业政策，大力推进“创业促就业、培训促就业、政策促就业”工程，市财政专门安排6000万元资金设立创业贷款担保基金，有力激发了全民创业的热情。全年新增城镇就业11.78万人，农村新增劳动力转移10.6万人，城镇登记失业率控制在4%以内。城市居民人均可支配收入和农民人均纯收入达到16800元、6200元，分别比上年增长13%和12%。

　　始终把保障群众基本生活作为政府义不容辞的责任，不断完善社会保障体系，全年财政安排养老金兜底、离退休人员医疗费、城镇居民医疗保险等社会保障支出37.68亿元，社会保险覆盖面进一步拓展，保障水平稳步提高，企业基本养老金、城乡居民低保标准均有了较大幅度提高。建立了针对城乡困难群众的三项救助制度，25万困难群众的看病、就医等基本生活困难得到缓解。投资2.9亿元解决了73万农村人口的饮水安全问题。市委、市政府为民办的九大类实事年度任务全面完成。

　　坚持城市发展为了人民、项目建设贴近民心，在财力并不宽裕的情况下实施了一系列住房保障工程，群众居住环境明显改善。投资1.9亿元对全市78个老居民小区进行了综合整治，近30万群众受益。完成20万平方米第三期经济适用房和100套廉租房建设任务，启动建设了第四期经济适用房、廉租房和120万平方米定销商品房，实现了对市区符合条件、申请廉租房租赁补贴低收入家庭的应保尽保。

　　科技教育等各项社会事业健康发展。科教兴市战略深入推进，全市新增省级高新技术企业20家、省级高新技术产品126个，专利申请量达5400件，矿大国家大学科技园总部基地开工建设，我市荣获“中国城市综合创新力五十强”和“中国最具创新绩效城市”称号。城乡义务教育阶段学生全部免除学杂费、书本费，初中毕业生升学率达95%，外来人员子女义务教育入学率达100%，职业教育和高等教育加快发展，市职教中心、幼儿高等师范学校新校区迁建顺利完成，徐州工程学院新校区开工建设。文化产业园列入第二批江苏省文化产业示范基地，镇标准文化站建设全部达标，徐州琴书等8个项目列入国家级非物质文化遗产名录。公共卫生体系日趋完善，80%的城市社区卫生服务机构建设实现标准化，70%以上的农民医疗问题能够在村镇解决。全年计划生育率为85%以上，出生人口性别比下降至120左右，为农村100万名育龄妇女提供了免费生殖健康服务。市县两级国民体质监测中心全部建成，105个乡镇和41个街道办事处建成“体育健身中心”，我市运动员荣获1枚奥运银牌、7枚残奥金牌。“平安徐州”、“法治徐州”创建深入推进，遍及城乡的监控网络基本形成，镇（街道）综合治理法治工作中心全面建成，应急处置能力进一步提升；全部刑事案件、八类案件、命案等案件数持续下降，我市跨入全省社会治安综合治理和平安建设工作先进市行列，荣获国家科技强警示范城市称号。安全生产形势保持稳定，食品药品等产品质量安全水平稳步提高。支援四川灾区抗震救灾和对口援建工作扎实有力，充分彰显了徐州人民有情有义的精神风貌，得到了省委、省政府和灾区群众的充分肯定。人防、民兵、民族宗教、民政、扶贫、档案、外事、侨务、台湾事务、统计、审计、物价、广播电视、无线电管理、新闻出版、气象、妇女儿童、青少年、关心下一代、老龄、残疾人等各项事业都取得了新的进展。

　　（五）机关作风建设深入开展，政府效率效能进一步提高。制度创新力度加大，AB岗工作制、限时办结制、“快速通道”制等制度逐步完善。加强政府部门权力运行内控机制建设，细化了行政职权目录和行政权力运行图，向社会公开了办事依据、责任主体等重要环节；行政检查、行政收费和行政处罚公开化试点工作逐步深化。强力推进政务提速，认真查纠违法设立行政许可、“中梗阻”、办事效率低下等问题，涉及重大项目建设和企业发展等审批事项的办理时限进一步压缩，四级便民服务网络进一步完善，所有镇和街道办事处的便民服务中心建成运行。严格落实党风廉政建设责任制和行政执法责任制，自觉接受市人大及其常委会的法律监督和工作监督，主动接受市政协的民主监督及社会各界的监督，廉政建设和民主法制建设进一步加强。

　　在充分肯定成绩的同时，我们也清醒地认识到，当前经济社会发展中还存在不少矛盾和问题。主要是：受国际金融危机的影响，我市工业经济增长速度呈现回落趋势，部分企业生产经营较为困难；房地产市场低迷对主城区财政收入的影响较大；资金、土地等要素制约依然紧张，部分项目推进较慢，大项目、好项目、龙头带动型项目偏少。加强节能减排的长效机制尚未根本形成，环境保护压力依然较大。城市就业形势趋紧，农民增收难度加大，一些事关群众切身利益的问题还没有得到根本解决。农村基础设施建设任务还相当艰巨，城市管理还存在着薄弱环节。机关作风和效率效能建设仍需进一步强化，一些干部不求精细、缺乏创新的工作作风还未彻底转变，少数部门的服务意识和工作效率还有待进一步提高。对这些问题，我们将高度重视，认真加以解决。

　　各位代表，过去的一年，在全球经济萎缩和国内经济环境趋紧的形势下，全市经济仍保持了较快发展速度，各项社会事业取得较为明显的进步，成绩来之不易。这是全市上下共同努力、开拓奋进的结果，是各位人大代表、政协委员和社会各界人士监督、支持与帮助的结果。在此，我代表市人民政府，向全市广大干部群众、人民解放军驻徐部队、武警官兵和公安民警，向各民主党派、工商联、人民团体和无党派人士，向热心支持徐州现代化建设的香港特别行政区同胞、澳门特别行政区同胞、台湾同胞、海外侨胞和各界人士，向投身徐州经济社会发展的国内外投资者和企业家，表示衷心的感谢并致以崇高的敬意！

　　　　　　　　　　　　　　　　　　　二、今年政府工作的主要任务

　　综观国际国内形势，2009年将是新世纪以来经济发展最为困难的一年，也是挑战和机遇并存的一年。国际金融危机的影响尚未见底，对全球经济的影响仍在进一步加深；我国经济运行中的困难增加，经济下行压力加大。随着各种不利影响由沿海城市向内地城市延伸，特别是由消费工业向材料工业、装备制造业和新兴产业扩散，我市将在更大层面上受到冲击。同时，也要清楚地看到，中央审时度势，果断决策，出台了一系列扩大内需、促进增长的重大举措，出手之快、出拳之重前所未有，必将对今年经济发展产生积极而深远的影响。尽管我们面临许多各地共有的压力，但是更面临特有的历史性发展机遇，省委、省政府关于加快振兴徐州老工业基地的意见，明确提出徐州加快振兴的目标定位、主要任务和扶持发展的政策措施，必将为我市今后的发展注入强大的动力；徐州正式纳入长三角经济圈，有利于我们主动承接上海经济圈和苏南先进地区的产业和资本转移，不断加速全市的对外开放。在这种形势下，我们必须始终坚持在解放思想中解决问题，在更新观念中创新思路，在应对危机中抓住机遇，变压力为动力，化不利为有利，以更大的决心和力度，牢牢把握发展主动权。

　　今年政府工作的总体要求是：坚持以邓小平理论和“三个代表”重要思想为指导，深入贯彻落实科学发展观和党的十七届三中全会精神，把保增长作为首要任务，积极应对各种困难和挑战，全面加快老工业基地振兴，强力推进招商选资，大力强化有效投入，积极推进农村改革发展，加大区域性中心城市建设力度，加强生态环境保护建设，下更大的力气改善民生民计，努力开创改革开放和现代化建设的新局面，为新一轮的跨越发展奠定坚实基础。

　　今年国民经济和社会发展的主要预期目标是：（1）地区生产总值增长13%。（2）财政一般预算收入增长16%。（3）全社会固定资产投资增长25%。（4）实际到帐注册外资和自营出口总额分别增长18%和15%。（5）社会消费品零售总额增长18%。（6）城市居民人均可支配收入增长11%，农民人均纯收入增长10%。（7）城镇登记失业率控制在4%以内。（8）万元GDP能耗下降5%，化学需氧量削减1.73%，二氧化硫排放量削减10.72%。（9）人口自然增长率控制在6‰以内。

　　围绕实现上述目标，我们将把扩大有效投入作为保发展、保增长、保民生、保稳定的关键举措，牢牢抓住国家扩大内需的机遇，以“两大中心”、“三大基地”和“四大产业”建设为主线，大力强化重大基础设施项目、重大产业项目、重大功能性项目和重大生态环境项目建设。全市计划安排重大项目130项，其中年度实施100项、总投资1331亿元、年内完成投资484亿元，前期推进30项、总投资558亿元。我们将按照“出手要快、出拳要重、措施要准、工作要实”的要求，确保重大项目建设取得明显进展，强力推动经济发展方式转变和经济结构战略性调整。

　　今年，我们将主要做好以下六个方面的工作：

　　（一）坚持新型工业化第一方略，加快转变经济发展方式。徐州老工业基地的振兴，关键在于工业的振兴。我们要把新型工业化放到更加突出的位置，全力以赴加以推进。

　　一是以扩大有效投入推动自主创新和结构调整。坚持用高新技术改造提升传统产业，大力发展高新技术产业和新兴产业，不断增加有效投入，强化自主创新，加速推动产业集聚、产业转型和产业升级。以增强徐工集团、卡特彼勒徐州公司、徐挖公司为代表的装备制造业核心竞争力为重点，加快推进工程机械主机产品和关键零部件开发，突出抓好徐工载重汽车、大功率发动机、大型履带起重机和铜山徐挖公司挖掘机、睢宁环宇公司工程机械等重大项目。强化食品产业层次提升，突出抓好维维集团食品产业园、徐州卷烟厂“十一五”技改、新沂鲁花食用油等重大项目，建设县域六大特色食品产业基地。大力发展光伏光电等新能源产业，加大对产业链高端和高新技术的攻关力度；继续实施“煤电冶金、煤电化工、煤电建材”三大产业链延伸工程。突出抓好中能硅烷气、艾德太阳能电池等光伏光电重大项目，罗特艾德、维斯塔斯等风电设备重大项目，贾汪徐矿资源综合利用电厂一期、彭城电厂三期、邳州徐塘电厂三期等电力重大项目，全力支持中能光伏、艾德太阳能等企业争创国家和省级技术中心。积极推进化工、医药、纺织、冶金等产业优化升级，重点抓好丰县丰成联碱、沛县大屯高精度铝板带、新沂恒艺服装加工、邳州沂州煤焦化、贾汪中联水泥二期、恩华医药工业园等一批重大项目。围绕重大项目建设的需求，要在坚持最严格的耕地保护制度和最严格的节约集约用地制度的前提下，用足用好采煤塌陷地治理、农村建设用地与城镇建设用地增减挂钩等各项政策，依法打击违法违规用地行为，强化重大项目用地保障，并积极破解项目建设的资金和报批等瓶颈制约，确保重大项目顺利推进。

　　二是继续大力推进招商选资。围绕增强发展后劲，强化产业链招商，抓好重点区域招商。要突出港台招商，强化欧美和日韩招商，按照主导产业发展方向，更大规模地承接国际和国内发达地区的产业转移，着力引进科技含量高、产出规模大、带动能力强的大项目好项目；切实做好已落户我市的世界500强、中国100强投资项目的延伸招商，不断增加后续投资项目。精心办好汉文化旅游节暨经贸洽谈会等综合性招商活动，中央和省大型国有企业、工程机械和专用车辆、物流等专题招商活动。完善招商绩效评估和考核机制，进一步提高项目工商注册率、资金到位率和开工投产率。

　　三是加快开发区和南北共建园区建设。继续加大向开发区放权力度，确保开发区具有相对独立的行政审批权和经济社会管理等职能。完善省级开发区产业规划，强化特色园区培育，加快环境基础设施建设，积极推进申报国家级开发区和出口基地工作，大力提升开发区项目承载力和产业集聚度。积极支持徐州经济开发区开展“创新推进年”活动，狠抓主导产业提档升级，加快建设国内一流的创新型开发区。扎实搞好南北共建园区工作，大力吸引苏南企业与我市企业合作开发接续替代产业，推进产业承接和共同发展。

　　四是强力推进节能降耗和控污减排。在电力、纺织、建材、化工等重点耗能行业和年耗标煤5000吨以上的重点耗能企业，组织开展节能专项行动，推广应用节电、节煤、节水等新技术，加快淘汰落后产能。综合采取法律、经济和行政手段，坚决关闭小化工、小水泥等重污染企业，进一步消除结构性污染。加强饮用水源地保护与管理，扎实推进截污导流工程和奎河黄桥、沛沿河李集桥等重点国控断面水质达标工作，大力防治秸杆焚烧，努力实现环境安全。

　　（二）加快农村改革发展，努力增强县域经济综合实力。农业是徐州在全省的亮点，也是我们的传统优势，要继续强化这一优势，落实强农惠农政策，进一步提升农业产业化、规模化和现代化水平，全面推进农村改革和发展。

　　一是着力提升现代农业发展水平。加强粮食综合生产能力建设，稳定种植面积，优化品种结构，不断改善农业基础设施条件，确保粮食生产稳定发展。深入推进农业结构调整，大力发展高效规模农业，重点建设10个万亩设施农业基地和10个规模养殖场，力争年内新增设施农业10万亩。全面提高农业产业化水平，依托十大优势主导产业，力争引进培育一批产业关联度大的农产品加工龙头企业，在各地集中力量办好一个农业科技示范园、一个农产品大市场和一个农副产品加工区。着力在粮棉油生产上通过种田大户加社会化服务实现规模经营，在高效经济作物生产上通过发展设施农业加专业合作实现规模经营，在畜牧水产业发展上通过养殖基地加龙头企业实现规模经营。坚持从源头上提高农产品质量，大力推行标准化生产、品牌化经营，加快实行对种植和养殖业生产、销售的全过程监管，确保食品安全。

　　二是积极推进农村各项改革和制度建设。要在规范和创新农村土地管理制度上取得新突破，在坚持农村土地承包关系长期不变的前提下，稳定和完善农村土地使用制度，引导农民按照“依法、自愿、有偿”原则流转土地承包经营权，并切实解决好被征地农民的就业、住房和社会保障问题。要在建立现代农村金融制度上取得新突破，大力创新农村金融体制，推进农村小额贷款组织和村镇银行试点工作，加快建立健全农村信贷机制和保险制度，发展各类适合农村特点和需要的新型金融组织。要在发展农民专业合作组织上取得新突破，鼓励种粮大户、农业科技人员等领办创办农民专业合作组织，年内创办农民专业合作社100家。要在完善农业支持保护制度上取得新突破，对土地出让收入、耕地占用税新增收入，切实按照政策规定的比例足额用于农村，逐步增加农民种粮补贴，完善农资综合补贴动态调整机制，持续加大财政对“三农”的投入力度，健全强农惠农的政策体系。

　　三是大力发展农村公共事业。组织实施农村人才、农民健康、为农服务、农村文化、农村环境和脱贫攻坚六大工程。加强农村人才队伍建设，加快培养农村实用型人才；巩固完善新型农村合作医疗制度，力争以县为单位农村社区卫生服务中心（站）覆盖率达95%以上；加快镇村两级综合服务体系建设和政策性农业保险业务拓展；积极推进文化惠民工程，实现镇标准文化站全面开放；突出抓好600个环境综合整治示范村建设；继续深化“双百村帮扶工程”，对返乡农民工进一步加大就业技能培训力度，努力使农民工返乡不失业。

　　四是深入推进城乡一体化进程。大力发展县域经济是统筹城乡发展和推进城乡一体化的关键所在，要进一步强化对县域经济发展的政策扶持和要素保障，加快县域工业化和城市化进程，推动县域经济增长高于全市平均水平。从统筹推进城乡建设规划、产业布局、基础设施、公共服务、就业一体化入手，集中力量抓好各地重点园区、重点乡镇、重点企业、重点项目，在工作上放手、政策上支持、资源上倾斜，进一步壮大县域经济实力。完善各县（市）城市总体规划，建设好40个特色鲜明的中心镇，推动新农村150个市级示范村提档升级，新建50个市级达标示范村。放宽县城、小城镇落户条件，加快县城“城中村”改造步伐，拓展城镇发展空间。构建城乡统一的人力资源市场，开展城乡统筹就业工作试点。统筹城乡基础设施建设，加快形成城乡统一的公共服务体系。继续加大“突破睢宁”工作的力度。在铜山县、贾汪区、九里区和徐州经济开发区建立市级统筹城乡协调发展区，探索推进城乡一体化的新途径。

　　（三）突出层次提升，着力推进服务业大发展。徐州服务业的基础条件好、发展潜力大，要以建设区域性商贸物流旅游中心为重点，因势利导，科学规划，加快发展，使其成为全市经济社会发展的重要增长极。

　　一是重点发展商贸物流旅游等现代服务业。根据服务业发展的基本趋势及我市现有产业基础、资源条件、市场潜力和比较优势，重点发展商贸、物流、旅游、商务服务、科技服务和房地产业，统筹发展文化教育、医疗卫生、金融保险、社区服务等其他服务业。在商贸流通业方面，集中力量推进中心商圈、新城区商圈和以京沪高铁商务区为核心的东部商业中心建设，加快构建市区“一主四副”五大商圈的总体布局；协调推进城乡消费连锁化、便利化，加快形成定位准确、层次分明的县域商贸流通发展体系。引导广大商业企业开展“‘五一’黄金周欢乐购”等大型让利促销活动，进一步增强对周边地区的吸引力，有效扩大消费需求。在现代物流业方面，重点规划建设徐州经济开发区生产物流、新城区生活物流、九里综合物流等五大物流基地，积极构建全国一级综合物流枢纽城市。在旅游服务业方面，坚持文化兴旅、商贸兴旅、交通兴旅等多元驱动，制订全市旅游业发展总体规划，重点建设一批重大旅游项目，积极推进大型游乐设施和野生动物园建设，进一步挖掘利用云龙山、云龙湖、微山湖、骆马湖、马陵山等山水旅游资源和两汉文化、书画文化、军事文化等历史文化资源，加强与周边城市的旅游协作与文化交流，努力建设区域性旅游中心城市。在商务服务业方面，积极发展总部经济、服务外包、电子商务、投资咨询、广告会展等新兴业态，尽快培育形成高端商务服务业集聚中心。在科技服务业方面，大力发展科技研发、工商设计、产品创意、教育培训等技术服务，加快构建科技服务业强市。在房地产业方面，全面贯彻落实国家和省关于促进房地产业发展的各项政策措施，进一步加大保障性住房建设力度、住房消费政策落实力度和房地产市场引导力度。稳步出台土地供应、金融支持、公积金保障等配套措施，增加保障性住房供给，控制低密度高档住宅用地供应量，对棚户区改造和新城区住房建设进行政策倾斜；积极鼓励居民购买自住性、改善性住房，放宽居民购买二套房贷款条件，降低二手房交易税费，支持普通商品住房消费，促进房地产市场平稳健康发展。

　　二是扎实推进现代服务业集聚发展。坚持规划引导，研究制定《徐州市服务业产业发展与布局规划》和《徐州市服务业集聚区发展专项规划》。重点依托中心城区、城郊结合部市场带、交通枢纽、重点开发区和科技园区，培育建设中央商贸、现代物流、科技创业、创意产业、商务服务和产品市场六大类现代服务业集聚区。积极引进国内外知名服务业企业和功能型、税源型、就业型服务业大项目，加快全市服务业结构调整和布局优化。

　　三是完善扶持服务业发展的各项政策。建立健全政策支持体系，重点在财政税收、融资环境、品牌培育、项目支持、集聚区建设等方面，力求政策创新和突破。继续加大财政支持力度，积极整合服务业专项资金，增加服务业发展引导资金额度。强化对服务业创业主体的贷款担保支持，进一步优化服务业发展的金融环境。支持服务业骨干企业、知名品牌实施标准化建设，尽快提升整体发展质量和水平。

　　（四）强化基础设施建设，提升区域性中心城市功能。徐州最大的优势是区域性中心城市，我们必须坚持不懈地把硬件建设好、把软件搞上去，真正把徐州这个中心城市做大做强做优。既要积极进取、尽力而为，又要稳妥推进、量力而行，全市城建重点工程分两批实施，从一月份开始推进一批，另一部分到六月份视情况再予以启动。

　　一是重点推进交通枢纽、商贸物流和旅游文化“三大中心”建设工程。徐州要强化区域性中心城市地位，必须抓好交通枢纽中心建设。高标准推进京沪高铁枢纽站工程，做好高铁站区的规划设计工作，年内完成站区村庄搬迁和大部分土地整理，启动基础设施建设。积极推进徐兰客运专线动迁征地等前期工作，力争开工建设。加快丰沛铁路、徐济高速公路江苏段、宿新高速公路徐州段建设步伐。完成火车站综合改造、淮西客运站枢纽改扩建等工程建设任务。加快推进东调南下等重点水利工程建设，积极争取实施湖西航道改造工程。加快观音机场二期扩建和亿吨大港项目前期工作，启动大郭庄机场搬迁前期论证。开工建设贾汪至徐州、新城区至铜山新区两条快速通道，推进连接徐海路与新城区的高新路建设，完成徐丰公路、城东大道综合整治。推进市区道路畅通和公交优先工程，抓好淮海路、中山路延伸工程，改扩建建国西路等７条道路，综合改造永安广场等７个道路交叉口，更换部分线路公交车辆。全力推进商贸物流中心建设。着力抓好苏宁综合商务广场、金鹰国际二期、中央国际商务广场、彭城北路商业广场、乐宾百货商城等中心商圈五大项目，协调推进滨湖公园商业步行街、汉之源商业步行街等商贸项目及一批重大物流项目。全面加快旅游文化中心建设。加快实施音乐厅、美术馆、百果园、吕梁山风景区、汉文化景区二期、龟山民间博物馆区等重大旅游项目。加强新沂窑湾古镇、丰县汉皇祖陵的风貌保护和深度开发，搞好丰县大沙河生态旅游观光带、邳州银杏园、睢宁下邳特色农业观光园、沛县微山湖旅游度假村、新沂瓦窑农业旅游风景区等农业生态旅游项目建设。

　　二是精心实施新城建设、山水景观、环境改善和民生实事工程。在新城建设工程方面，在保证规划馆、档案馆、科技馆、新闻中心等重大项目如期竣工的同时，有序推进公交首末站、污水处理厂、垃圾中转站等市政配套设施建设，启动中小学校、医院、商业等一批公共配套项目建设；着力打造拖龙山自然生态山体公园，实施顺堤河、肖庄河、大韩河等河道整治及景观绿化工程；稳步推进房地产开发和现代物流项目建设。在山水景观工程方面，重点实施市民广场、泰山凤凰山山景公园、白云山公园二期、滨湖公园景观改造等项目，继续推进第三期市区山地绿化造林，力争年内市区新增改造绿地1000公顷，全市植树造林10万亩。在环境改善工程方面，加快推进采煤塌陷区生态修复，组织实施西区污水处理厂、荆马河污水处理厂二期、三八河污水处理厂扩建、丁万河污水处理厂、小沿河清淤等项目。在民生实事工程方面，重点实施经济适用房与廉租房、定销房“三房”建设，着力抓好棚户区改造、居民小区整治、道路改扩建、环卫基础设施建设等项目。年内改造棚户区70万平方米，开工建设120万平方米定销商品房，完成1500套经济适用房建设任务，建设500套廉租房，投资1.95亿元对51个、435万平方米的老居民小区进行整治。

　　三是继续强化城市规范化、科学化管理。在巩固保洁、绿化市场化管理成果的基础上，全面推行城市管理的规范化、标准化、长效化措施，建立城市管理快速反应机制，逐步将户外广告设置、公厕管理等推向市场，年内确保市区主干道大型户外广告设置市场化运作率达95%以上。正确处理好城市管理中堵与疏的关系，抓好12条“严管街”、23条重点道路、6个城市出入口的环境综合治理。加快城市电子监控资源整合步伐，建成城市管理数字化监控平台。

　　（五）深入推进改革开放，为又好又快发展注入新的活力。以深化改革逐步消除体制机制障碍，以扩大开放加快经济国际化进程，争创新的竞争优势。

　　一是继续深化各项改革。进一步完善市属企业结构和法人治理结构，健全国有资本经营预算制度，加大骨干企业上市工作推进力度，加快徐工集团整体上市步伐。有序推进事业单位机构改革。加强财政体制改革，做好增值税转型、燃油税征收等税费改革工作，健全市、区财力与事权相匹配的财政体制，深化省直管县的财政管理体制改革，提高公共财政服务水平。深化金融改革，努力吸引外地股份制商业银行在徐州设立分支机构。

　　二是进一步扩大对内对外开放。抓住苏北融入长三角经济圈和沿海开发上升为国家战略的机遇，大力优化投资环境，积极扩大开放领域。全面落实国家和省促进外贸发展的一系列扶持政策，在出口信用保险、企业退税等方面加大对出口企业的帮扶力度，推进工程机械、光伏光电、农产品及轻工产品等出口基地建设，进一步优化出口产品结构，引导出口企业积极开拓东盟、中亚、中东和南美市场，努力扩大欧美市场份额。加大外经企业项目开发合作力度，拓展与我国中西部地区的经济联系，加快传统资源能源型产业对外投资步伐。进一步加大招才引智工作力度，重点引进30名“领军型”海外留学归国创业人才和100名高层次创新创业人才。

　　三是强化对民营经济的政策扶持。全面落实提振中小企业发展的各项措施，鼓励民营企业强化自主创新、实施品牌战略，不断提升产业集聚度和核心竞争力。针对部分中小企业面临的经营困难，积极构筑中小企业服务、融资担保和行业协会平台，建立中小企业发展专项资金，综合运用创业投资基金、风险投资基金，切实缓解中小企业的资金难题。

　　（六）大力改善民生，扎实推进和谐社会建设。作为人民的政府，越是在困难的时候，越要关注民生，越要关心困难群众，越要把群众的安危冷暖放在心上。要继续大力推进“三业六有”工程建设，不断提升社会事业发展水平，切实让广大人民群众得到更多实实在在的利益。

　　一是强化富民惠民的制度建设。进一步完善小额担保贷款、税费减免、职业技能培训补贴、创业风险担保基金等制度，优化创业环境，推动全民创业。实行更加积极的就业政策，完善就业援助制度，确保全年新增就业7.5万人，失业人员实现再就业3.5万人，城镇登记失业率控制在4%以内。加快完善各项社会保障制度，确保城镇失业、养老、医疗三大保险参保率均达到98%，最低生活保障做到应保尽保。在义务教育学校实施绩效工资，确保义务教育教师平均工资水平不低于当地公务员平均工资水平。

　　二是全力办好事关群众切身利益的实事项目。坚持注重民生、贴近百姓，坚持公共财政支出进一步向农村和农民倾斜、向低收入群体倾斜，重点在加强社会保障、生态环境建设、教育卫生服务设施建设、居住和消费环境提升、出行条件改善、公共服务设施完善、新农村建设、社会治安管理等方面，精心组织实施八大类实事项目，切实解决好人民群众最关心、最直接、最现实的衣食住行问题。

　　三是统筹发展各项社会事业。加快建设省级智能化工程机械和高新技术产业基地，不断完善市、县（市）区两级科技创新平台体系，力争组织实施国家和省级高新技术、科技支撑、科技攻关等8个项目，认定高新技术企业（产品）60个，高新技术产业产值达260亿元。推进教育均衡化发展，加快教育现代化进程，力争全市80%以上的县（市）区完成教育现代化创建任务；加强高等教育和职业技能教育结构调整，进一步强化徐州职业教育的特色，抓好三十四中新校区、第二职业中学艺术中心、徐州特教中心康复中心等工程建设，完成徐州工程学院新校区一期工程建设任务，支持徐州生物工程学校申办徐州农业职业技术学院、徐州幼儿高等师范学校申办徐州幼儿师范高等专科学校。

　　切实抓好其他社会事业。大力发展文化产业，实施文化精品工程，进一步打造徐州的特色文化品牌，加快文化大市向文化名市转变；围绕庆祝新中国成立60周年，精心组织“欢乐国庆嘉年华”等系列活动，营造欢乐祥和的节庆氛围。进一步健全社区卫生服务体系，力争新型农村合作医疗以县为单位参合率稳定在95%以上、农民住院费用实际补偿比例达到40%以上；继续抓好农村改厕工作，完成20万户农村卫生改厕任务；大力实施饮水安全工程，解决好99万农村人口饮水安全问题。进一步完善城乡体育网络，加快3.5万人规模的新体育场工程建设，积极申办江苏省第十八届运动会。高度重视人口和计划生育工作，继续稳定低生育水平，启动免费自愿婚检工程，促进出生人口性别比趋于平衡，提高出生人口素质，全面实施“新农村新家庭”计划。强化物价监控和市场监管，抓好产品质量和食品药品安全。进一步抓好人防、民兵、双拥、民族宗教、档案、外事、侨务、台湾事务、统计、审计、广播电视、无线电管理、新闻出版、气象、妇女儿童、青少年、关心下一代、老龄、残疾人等方面的工作，促进各项社会事业协调发展。

　　四是全力维护和谐安定的社会环境。积极畅通民意诉求渠道，严格落实信访工作责任制，认真解决群众反映的突出问题。加强社会治安综合治理，全面落实大防控、大调解和基层基础“三大建设”的各项措施，深入开展矛盾纠纷排查化解工作，严厉打击各种违法犯罪活动，精心打造“平安徐州”、“法治徐州”，全力维护社会稳定。健全应急管理体制，进一步提高预防和处置突发事件的能力。高度重视安全生产，坚决遏制重特大事故的发生。加快推进对口支援四川省绵竹市富新镇地震灾后恢复重建工作，确保高标准、高质量完成年度援建任务。

　　　　　　　　　　　　　　　　　　　三、加强政府自身建设

　　在宏观经济形势复杂多变的大背景下，在加快徐州老工业基地振兴的历史性机遇面前，我们必须进一步加强自身建设，努力以政府工作的新作为开创加快振兴的新局面。

　　一是大力激发奋发有为、攻坚克难的精神状态。无论宏观形势多严峻，只要精神不滑坡，办法总比困难多。要大力引导广大干部进一步振奋精神，始终保持锲而不舍、坚持不懈的工作干劲，坚决摒弃事不关己、得过且过的消极思想，切实做到对群众充满感情、对工作充满热情、对事业充满激情、对挑战充满豪情。要在全市开展服务企业、服务创业、服务就业的“三服务”活动，促进经济平稳健康发展。要进一步完善机关效能评议、考核和监督的长效机制，严肃查处有令不行、有禁不止和不作为、乱作为等行为，坚决做到失职必罚、渎职必究，以更大的力度破除“中梗阻”、废除“潜规则”，在全市上下营造人人思进、处处争优，聚精会神搞建设、心无旁骛谋发展的生动局面。

　　二是着力打造勇于创新、敢于负责的政务环境。因循守旧没有出路，畏首畏尾难以发展。我们必须坚持激励与鞭策相结合，建立有利于创新的动力机制，倡导大胆创新的工作作风，提高各级干部抢抓机遇、破解难题、高效落实的能力。开展提高“政府执行力”建设年活动，推进政府决策、执行、监督等方面的改革工作，倡导敢于负责、勇于担当的工作作风，坚决纠正精于关系、疏于干事的不良风气，以非常时刻非常之举、关键时期超常之策推动政府工作的开展。

　　三是合力塑造勤政、廉政和优政的政府形象。要以制度设计为根本，健全权力运行内控机制。强化行政服务中心建设，推进各部门行政许可事项入驻到位、人员授权到位、“一站式”办理到位，力争入驻率和窗口现场办结率分别提高至85%和80%。全面推行政务公开，从制度上建立与完善“零障碍、低成本、高效率”、“急事快办、特事特办、难事巧办”和“有报必批、有批必办、有办必果、有果必报”的政务绿色通道。实行行政“首长负责制”，切实加强对领导干部特别是主要领导干部的监督，推进干部岗位交流。以改革创新的精神抓好《建立健全惩治和预防腐败体系2008－2012年工作规划》的落实，坚持惩防并举、重在建设，在坚决惩治腐败的同时，更加注重治本，更加注重预防，更加注重制度建设，深入开展“勤廉徐州”创建活动，大力提高各级干部勤政、廉政、优政的综合素质，真正建立起“奖勤罚懒、奖廉惩腐、留优淘劣”的长效机制。强化厉行节约，市级单位实行公务购车和用车经费、会议经费、公务接待费用、党政机关出国（境）经费预算“四个零增长”。以外部监督为动力，主动接受市人大及其常委会的法律监督和工作监督、市政协的民主监督、新闻舆论监督和人民群众监督，积极加强与各民主党派的对口联系，促使广大政府公务员勤勤恳恳做事、干干净净做事、尽善尽美做事，牢固树立为民、清廉、务实的良好形象。

　　各位代表，面对新形势，面临新机遇，做好今年各项工作任务艰巨、意义重大。让我们在党的十七大和十七届三中全会精神指引下，在省委、省政府和市委的正确领导下，抓住机遇、攻坚克难，开拓进取、扎实工作，为加快徐州老工业基地振兴、推进又好又快发展而团结奋斗，努力在新一轮发展中实现新的跨越！
